# Oxycodone: A Pain‐Relieving Agent With Cardioprotective Properties Against Myocardial Ischemia–Reperfusion Injury

**DOI:** 10.1155/cdr/6182342

**Published:** 2026-02-13

**Authors:** Mehdi Dehghani Firoozabadi, Behrang Nooralishahi, Fatemeh Rezaei-Tazangi

**Affiliations:** ^1^ Tehran Heart Center, Cardiovascular Diseases Research Institute, Anesthesiology, Critical Care and Pain Research Center, Tehran University of Medical Science, Tehran, Iran, tums.ac.ir; ^2^ Department of Anatomy, School of Medicine, Fasa University of Medical Sciences, Fasa, Iran, fums.ac.ir

**Keywords:** cardioprotective, myocardial ischemic–reperfusion injury, opioid, oxycodone

## Abstract

In the realm of cardiovascular diseases, myocardial ischemia–reperfusion injury (MIRI) is known as one of the main life‐threatening conditions with significant morbidity and mortality. Although some therapeutic approaches, like pharmacological treatments and ischemic preconditioning, have been suggested for MIRI, offering a functional approach with high effectiveness for MIRI remains challenging. Recent studies have indicated that oxycodone, a semisynthetic opioid used to improve acute to chronic pain, may be cardioprotective against MIRI. The current experimental evidence showed that these protective influences can stem from the ability of oxycodone to regulate apoptosis, inflammation, and oxidative stress, as well as promote cardiovascular parameters (e.g., infarct size, cardiac function, and endothelial integrity). Hence, the main purpose of this narrative review study is to summarize and discuss the anti‐MIRI potential of oxycodone with a mechanistic insight. This narrative review was also conducted to hold promise for researchers and clinicians to consider and assess its clinical capacity for subjects suffering from this challenging disease.

## 1. Introduction

Acute myocardial infarction (AMI) is known as one of the main reasons for death around the world, which mainly stems from ischemic heart disease [1, 2]. AMI is responsible for a notable all deceased cases globally, with a considerably higher incidence in the male sex than the female [3, 4]. Although well‐timed reperfusion therapy is a common strategy for AMI treatment, it has been revealed that the fast restoration of the coronary bloodstream is able to exacerbate myocardial injury, resulting in myocardial ischemia–reperfusion injury (MIRI), which has a high mortality and morbidity [5–8]. MIRI, for the first time, was introduced by Jennings et al. in 1960. It is denoted by cellular swelling, contraction of myofibrils, and sarcolemma impairment in the myocardium after a period of ischemia followed by reperfusion [9]. The pathophysiology of MIRI involves different cellular and molecular processes, including intracellular calcium overload, cell death (autophagy, apoptosis, necrosis, necroptosis, and ferroptosis), oxidative stress, mitochondrial damage, inflammatory reactions, and impairment in the function of endothelial cells [10–16]. Potential therapies for MIRI include pharmacological treatment, ischemic preconditioning, and the use of medical gases or vitamin therapy, which could significantly help experts develop strategies to inhibit ischemia–reperfusion injury [17, 18]. However, an effective strategy for preventing and treating MIRI has not yet been suggested. Ergo, managing this cardiovascular problem is of great importance in improving patient outcomes [19, 20]. In recent years, several documents have been published accentuating the cardioprotective role of oxycodone against some cardiovascular conditions, for example, lipopolysaccharide (LPS)‐caused myocardial injury through different mechanisms like repressing oxidation, programmed cell death (pyroptosis), and inflammation, as well as heart failure [21–24]. Oxycodone is defined as a semisynthetic opioid analgesic originating from the opium alkaloid thebaine. It has been offered to alleviate pain from moderate to severe degrees following an operation by the World Health Organization (WHO) [25]. Oxycodone has been effectively utilized for pain relief postoperatively for many years in diverse regions, such as Canada and Northern Europe [26, 27]. Interestingly, some recent experimental research has investigated the possible cardioprotective role of oxycodone in MIRI from cellular and molecular viewpoints and reflected hopeful results. Hence, the purpose of this narrative literature review is to summarize and abate data relevant to the functionality of this semisynthetic opioid against MIRI with a mechanistic insight.

## 2. MIRI and Pathogenesis

**Figure 1:**
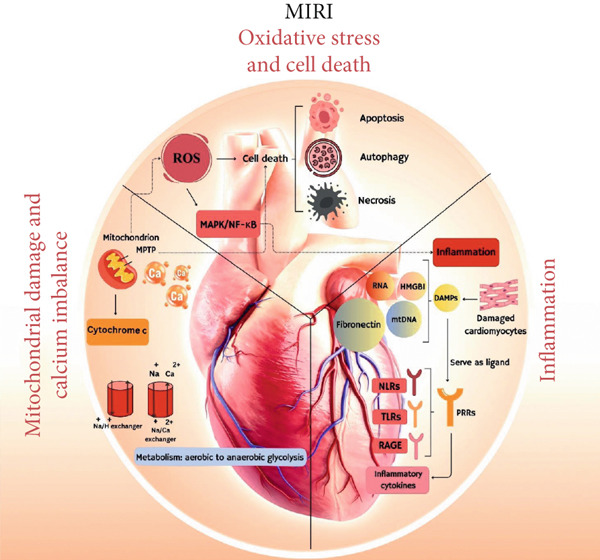
The role of processes related to inflammation, oxidative stress, cell death, mitochondrial damage, and calcium imbalance in the pathogenesis of myocardial ischemia–reperfusion injury (MIRI).

Till now, the pathological processes leading to MIRI have not been completely discovered [28]; however, the role of some agents has been highlighted (Figure 1).

### 2.1. Oxidative Stress and Cell Death

Reactive oxygen species (ROS), encompassing hydrogen peroxide (H_2_O_2_), superoxide, lipid peroxides, singlet oxygen, alkoxy radicals, H_2_O_2_, and hydroxyl and hydroxyl radicals, are known as small reactive agents engaged in the orchestration of different biological processes and cellular functions [29, 30]. At low to moderate levels, ROS functions as a signaling molecule, whereas its unmanaged high levels can yield free radical damage related to functional and structural changes in proteins, DNA, and lipids [31, 32]. During ischemia–reperfusion, particularly during reperfusion, the levels of ROS are elevated because of several mechanisms, for instance, the formation of xanthine oxidase, the neutrophil respiratory burst, and a failure in the mitochondrial electron transport chain [33]. ROS can damage cellular and molecular components (proteins, DNA, and lipids), resulting in cell death in the forms of apoptosis, autophagy, and necrosis [34]. Apoptosis and autophagy are two main forms of physiological or programmed cell death, which can also take place in pathological situations. Autophagy is described as a self‐degradative process critical for retaining the balance of energy in specific conditions. It exerts a housekeeping action by virtue of eliminating aggregated or misfolded proteins, destroying intracellular pathogens, and removing damaged organelles [35–37]. Necrosis is assumed to be an unprogrammed type of cell death that does not commit to the highly controlled and regulated intracellular programmed cell death like apoptosis. Dissimilar to apoptosis, necrotic cell death is an accidental and passive kind of cell death as a result of environmental factors, causing uncontrollable secretion of inflammatory cellular agents [38, 39]. ROS can also trigger multiple signaling pathways, such as the MAPK/NF‐*κ*B (nuclear factor kappa B) pathway, which intensifies apoptosis and inflammation. Moreover, oxidative stress participates in the opening of the mitochondrial permeability transition pore (MPTP) and calcium overload, which collectively are critical phenomena leading to cardiomyocyte death [34, 40–42].

### 2.2. Inflammation

Consequent to notable cardiomyocyte loss following ischemia and collateral damage conferred by reperfusion, damage‐associated molecular patterns (DAMPs) from the infarcted myocardium are released [43]. Released DAMPs comprise nuclear (e.g., HMGB1), cytosolic (e.g., RNA), extracellular matrix (e.g., fibronectin), mitochondrial (e.g., mtDNA), and contractile (cardiac myosin) components of the cardiac muscle [44]. DAMPs function as ligands for pattern recognition receptors (PRRs) such as NOD‐like receptors (NLRs), Toll‐like receptors (TLRs), and receptor for advanced glycation end products (RAGE). These receptors are broadly expressed in the heart and play a pivotal role in ischemia/reperfusion (I/R) injury by signaling in different cell types. The binding of DAMPs to PRRs elevates the expression of proinflammatory cytokines and chemokines, which confers sterile inflammation and provokes the innate immune system–related cells to the injured region [44–46]. RAGE and TLR2/4 trigger the NF‐*κ*B signaling pathway and instigate the NLR family pyrin domain containing 3 (NLRP3) inflammasome, whereas TLR3/9 triggers the cyclic GMP‐AMP synthase‐stimulator of interferon genes (cGAS‐STING) pathway and NF‐*κ*B, potentiating a type 1 interferon response [47]. Cardiac tissue–specific cells, comprising cardiomyocytes, macrophages, and fibroblasts, secrete inflammatory factors, such as tumor necrosis factor‐*α* (TNF*α*), interleukin (IL)‐1*β*, C‐C motif chemokine ligand 2 (CCL2), and IL‐6, which initiate a chemotactic response to attract myeloid cells [48–51]. Among these, IL‐1*β* worsens tissue injury by decreasing cardiomyocyte contractility, postponing fibroblast repair, and overexpressing the expression of adhesion molecules, which is further compounded by endothelial dysfunction [52, 53]. Particularly, the endothelium impairs its cell junctions and raises the expression of selectins and other cell adhesion molecules to allow leukocyte activity in the damaged tissue [54]. On the other hand, fibroblasts augment these inflammatory reactions by releasing GM‐CSF and some chemoattractants like C‐X‐C motif chemokine ligand 1 (CXCL1), CCL2, and CCL7, potentiating myeloid cell recruitment and crisis‐driven hematopoiesis [44]. DAMP release in the time of IR activates PRR signaling, provoking inflammation and leukocyte activation in the heart. Notably, oxidative stress and inflammation exacerbate each other in MIRI. Neutrophils and other leukocytes recruited to the injured myocardium generate additional ROS via the respiratory burst, amplifying oxidative damage. In turn, ROS activate proinflammatory transcription factors such as NF‐*κ*B, upregulating cytokines and adhesion molecules that further drive inflammatory cell recruitment. This vicious cycle worsens myocardial injury beyond the initial ischemia [7, 55, 56].

### 2.3. Mitochondrial Damage and Calcium Imbalance

In normal physiological conditions, the Na^+^/H^+^ exchanger (NHE) in the cell membrane plays a critical role in sustaining the balance between sodium (Na^+^) and hydrogen (H^+^) ions across the intracellular and extracellular spaces. Meanwhile, the Na^+^/Ca^2+^ exchanger (NCX) present in the cell membrane acts in order to keep intracellular calcium (Ca^2+^) levels [57, 58]. In the ischemic condition, the myocardial metabolism shifts from aerobic to anaerobic glycolysis, releasing H^+^ and lactic acid [59, 60]. Subsequent to this accumulation, the intracellular pH decreases, giving rise to the NHE to import Na^+^ ions and export H^+^ ions. This phenomenon, in turn, triggers the NCX, which expels excess Na^+^ and allows extracellular Ca^2+^ to enter the cell [61, 62]. During reperfusion, promoted NHE activity further produces intracellular H^+^, yielding a rapid reduction in intracellular pH [63]. Reperfusion of heart tissue that encompasses mitochondria damaged by a prior period of severe ischemia can trigger early mitochondrial injury. This injury is determined by the excessive formation of ROS and an imbalance in calcium regulation. As a result, there is abnormal permeation, swelling, and mitochondrial disruption, largely in light of the opening of MPTP [64, 65]. Following reperfusion and mitochondrial membrane potential (MMP) recovery, Ca^2+^ overload is transported into the mitochondria, thus elevating ROS levels, disturbing mitochondrial quality control (MQC), releasing cytochrome C (cyt C), and actuating MPTPs. These occurrences can justify myocardial cell death by autophagy, necroptosis, and apoptosis [42, 66–77]. Thus, mitochondrial dysfunction not only results from I/R but also amplifies injury: damaged mitochondria release prodeath factors (e.g., cyt C) and generate excess ROS, while impaired ion homeostasis leads to Ca^2+^ overload—together triggering cell death [7, 13, 55].

## 3. Opioid Receptors in the Cardiovascular System

Mu (*μ*), delta (*δ*), and kappa (*κ*) opioid receptors are expressed in the heart and vasculature, where they modulate cardiac physiology. These G‐protein‐coupled receptors are present on cardiomyocytes, nerve terminals, and vascular cells in the cardiovascular system [78–80]. Opioid receptor activation in cardiac tissue can induce membrane hyperpolarization and enhance vagal effects, leading to bradycardia, peripheral vasodilation, and hypotension [80]. *κ*‐ and *δ*‐opioid receptor stimulation in cardiomyocytes also exerts direct negative inotropic and lusitropic effects (reduced contractility and relaxation speed) by diminishing intracellular Ca^2+^ transients [23, 81], whereas *μ*‐opioid receptors have minimal direct impact on contractility [23]. Importantly, all three receptor subtypes trigger prosurvival signaling pathways within the myocardium. Opioid receptor agonism can activate the reperfusion injury salvage kinase (RISK) pathways (e.g., PI3K/Akt and ERK/MAPK) and other cardioprotective cascades, promoting mitochondrial K‐ATP channel opening and inhibiting mPTP opening. Endogenous opioids (enkephalins, endorphins, and dynorphins) and opioid drugs thus can induce “opioidergic conditioning,” wherein *δ*‐ and *κ*‐receptor activation, particularly, has been shown to reduce infarct size and arrhythmias during ischemia–reperfusion [78]. In summary, the opioid receptors distributed in cardiac and vascular tissues play a significant role in regulating heart rate, vascular tone, contractility, and cellular survival pathways in the context of cardiac.

## 4. Oxycodone and MIRI

**Figure 2:**
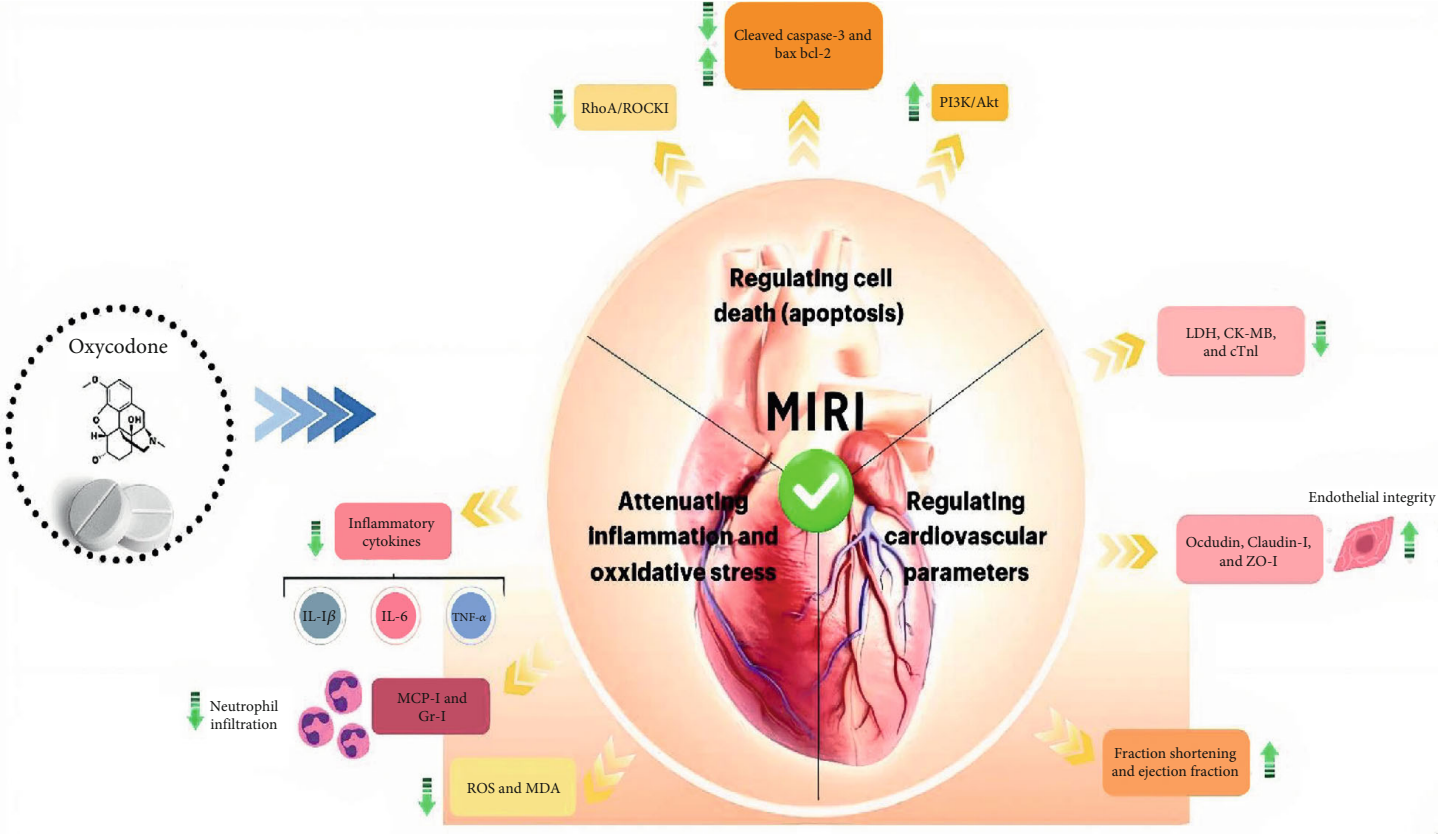
Cardioprotective mechanisms of oxycodone against myocardial ischemia–reperfusion injury (MIRI).

Recently, some experimental evidence has highlighted the importance of oxycodone therapy in conferring protection against MIRI through different cellular and molecular mechanisms (Figure 2 and Table 1).

**Table 1:** Documents related to oxycodone therapy for myocardial ischemia–reperfusion injury (MIRI) in vitro and in vivo.

**Dose**	**Route**	**Duration of reperfusion**	**Effect/mechanism(s)**	**Targeted signaling pathways/receptors**	**Model (in vitro/in vivo)**	**Ref.**
0.5 mg/kg	Intravenously (femoral vein)	2 h	Repressing apoptosis and regulating cardiac parameters (e.g., CK‐MB and cTnI)	RhoA/ROCK1 signaling pathway	In vivo	[82]
0.5, 1, and 1.5 ng/mL and 0.5 mg/kg	Intravenously	2 h	Repressing apoptosis, decreasing inflammatory cytokines (e.g., TNF‐*α*, IL‐6, and IL‐1*β*), promoting endothelial integrity, decreasing ischemic area, and potentiating myocardial function	SIGMAR1	In vitro and in vivo	[79]
0.1 mM and 0.5 mg/kg	Intravenously	2 h	Decreasing ischemic area, regulating cardiac parameters (e.g., cTnI, CK‐MB, and LDH), deceasing ROS formation and MDA levels, and repressing apoptosis	AMPK signaling pathway	In vitro and in vivo	[83]
1 ng/mL and 0.3 mg/kg	Intravenously	2 h	Decreasing myocardial infarct size, promoting cardiac function, attenuating apoptosis, and repressing oxidative stress	PI3K/Akt signaling pathway	In vitro and in vivo	[84]

### 4.1. Oxycodone Regulates Apoptosis in MIRI

Oxycodone has a pivotal role in attenuating apoptotic‐related pathways in MIRI by regulating apoptotic and antiapoptotic agents. Regarding this issue, experimental evidence has revealed that oxycodone has a protective role in the heart tissue of animal models of MIRI by elevating the expression of Bcl‐2, p‐PI3K, and p‐Akt and decreasing the expression of Bax and cleaved caspase‐3 genes [82, 84]. The B cell lymphoma (Bcl)‐2 family proteins have a key role in adjusting mitochondrial outer membrane permeability and thus monitoring apoptosis. Some proteins, like Bcl‐XL and Bcl‐2, serve as antiapoptotic agents, while Bcl‐2‐associated X (BAX), Bcl‐2 antagonist/killer 1 (BAK), and Bcl‐2‐associated agonist of cell death (BAD) have been known for their apoptotic effects [85]. The increase in phosphorylated PI3K (p‐PI3K) and phosphorylated Akt (p‐Akt) reflects triggering the PI3K/Akt signaling pathway, which is an important signaling pathway for promoting cell survival and repressing apoptosis [86]. Besides this pathway, the Ras homolog gene family member A (RhoA)/Rho‐associated coiled‐coil containing protein kinase 1 (ROCK1) signaling pathway is another molecular tool of oxycodone for making protection against MIRI by affecting apoptotic and antiapoptotic agents [82]. The RhoA/ROCK1 axis has a regulatory role in calcium homeostasis, cytoskeletal dynamics, and apoptotic pathways [87, 88]. Thus, it seems that the PI3K/Akt and RhoA/ROCK1 signaling pathways can be among suitable molecular targets for therapeutic purposes of this cardiovascular condition due to their potential for affecting apoptotic‐related pathways.

### 4.2. Oxycodone Regulates Inflammation and Oxidative Stress in MIRI

Oxycodone reduces the inflammatory reactions in the myocardial tissues of animals suffering from MIRI. In relation to this subject, the research of Ji et al. has demonstrated that oxycodone administration reduces and/or attenuates the levels of proinflammatory cytokines, including IL‐1*β*, IL‐6, and TNF‐*α*, inflammatory‐related signaling pathways (e.g., NF‐*κ*B signaling pathway), as well as neutrophil infiltration, as evidenced by decreased expression levels of monocyte chemoattractant protein‐1 (MCP‐1) and granulocyte‐1 (Gr‐1) in the serum and heart tissues of rats with MIRI [79]. The mentioned inflammatory factors have a pathogenic role in cell damage during MIRI conditions [89, 90]. Also, MCP‐1 and Gr‐1 are key indicators for neutrophil infiltration in this illness [91].

This drug is also able to regulate oxidative stress and sustain mitochondrial function in ischemic reperfusion injury of the heart tissue by declining ROS production and malondialdehyde (MDA) level, as a marker of lipid peroxidation, as well as maintaining MMP in H9c2 cells that underwent oxygen‐glucose deprivation/reoxygenation (OGD/R) injury [83]. Jiang et al. provided evidence that these results are related to the ability of oxycodone to activate the AMP‐activated protein kinase (AMPK) signaling pathway, as shown by elevating the phosphorylation of AMPK and the expression of PGC‐1*α* and SIRT1, vital modulators of oxidative stress and mitochondrial function [83, 92].

### 4.3. Oxycodone Promotes Cardiac Function and Attenuates Necrosis in MIRI

It has been confirmed that oxycodone can decrease myocardial infarct size and cardiac function by elevating the fraction shortening and ejection fraction in MIRI established in rats [84, 93, 94]. It can also elevate endothelial integrity in the disease model by promoting the expression of tight junction proteins, such as Occludin, Claudin‐1, and Zona occludens 1 (ZO‐1) [79]. In addition, some cardiac injury markers have been improved by oxycodone. It has been addressed that the serum levels of lactate dehydrogenase (LDH), creatine kinase‐myocardial band (CK‐MB), and cardiac Troponin I (cTnI) in following MIRI in animal models are decreased. The current documents accentuate the beneficial role of oxycodone in lowering the levels of these serum biochemical factors in rats who underwent MIRI, indicating the possible function of oxycodone in alleviating myocardial necrosis in this disease [82, 84].

A significant index of necrotic cells is the permeabilization of the plasma membrane, which can be detected in tissue culture media by assessing the levels of LDH, a key enzyme involved in gluconeogenesis and glycolysis [95, 96]. Another factor related to myocardial necrosis is CK‐MB, which is a type of myocardial enzyme that is chiefly found in the cytoplasm of cardiomyocytes and is a valid index for myocardial injury. During ischemia–reperfusion injury, cardiomyocytes undergoing necrosis release CK‐MB into the blood flow [97, 98]. Moreover, cTnI is named a gold standard biomarker for the diagnosis of AMI since it is specifically present in the myocardium and is secreted from the necrotic tissue of the heart [99].

### 4.4. Oxycodone and Pharmacological Considerations

The US Food and Drug Administration (FDA) approved oxycodone for the management of chronic or acute moderate‐to‐severe pain once opioid drugs seem to be appropriate and other pain relief approaches are insufficient [100]. However, there are some concerns regarding its applications for pharmacological purposes. Oxycodone is categorized as a controlled drug owing to its capacity for addiction. Its side effects are similar to those of other opioids, with constipation being the most prevalent adverse effect [101, 102]. Other possible complications include bradycardia, diaphoresis, respiratory depression, and abdominal pain [103–105].

Scientific evidence has revealed that this issue is increasingly observed in the United States [106, 107]. It is reported that the increased accessibility to opioids such as oxycodone has caused elevated mortalities. It is worth mentioning that most cases of oxycodone‐associated deaths are attributed to subjects who did not possess a valid prescription for it [108]. Emerging evidence suggests that the cardiac side effects of oxycodone are possibly mediated by suppression of the cardiac Nav1.5 sodium channel, which serves an indispensable role in the fast depolarization of cardiac cells. In this regard, it has been reported that this semisynthetic opioid changes cardiac electrical activity, for example, in long QT syndrome, which *conceivably* stimulates sudden cardiac arrest [109, 110]. Oxycodone may also cause other side effects related to oxycodone overdose, including hypertension, muscle flaccidity, and miosis. Its usage is also associated with acute hepatic injury, particularly in combination with high doses of acetaminophen [111, 112].

It is stated that oxycodone at concentrations of > 0.2 g/kg can participate in exerting toxicity; however, concentrations above 0.3 g/kg may not have toxic influences in a tolerant person [113]. In addition to the mentioned points, it has to be said that oxycodone can also have interactions with other drugs, leading to changes in its effectiveness. For instance, the coadministration of oxycodone with cytochrome P450 3A4 (CYP3A4) inhibitors (e.g., ritonavir, ketoconazole, and erythromycin) elevates the plasma levels of oxycodone, boosting opioid impacts [114]. On the other hand, its couse with CYP3A4 inducers, such as phenytoin, carbamazepine, and rifampin, reduces the therapeutic action of this opioid by decreasing its plasma levels [115].

Overall, the consumption of oxycodone with other drugs can be dangerous, mainly through two different mechanisms [108]:

1. Pharmacodynamic interactions (PDIs): This interaction, which confers a remarkable risk, comprises the concurrent use of opioids with other central nervous system (CNS) depressants like benzodiazepines. The combined pharmacodynamic impact leads to a promotion of respiratory depression, substantially elevating the possibility of a lethal outcome [116, 117].
2. Pharmacokinetic interactions (PKIs): The metabolism of oxycodone is dependent on certain hepatic enzymes. The concurrent consumption of drugs that repress these enzymes can cause increased systemic concentrations of this opioid, thus augmenting its toxic influences [118–120].

### 4.5. Comparison of Key Experimental Studies

Recent studies examining oxycodone′s cardioprotection have employed varied experimental designs (Table 1). For example, Xie et al. [82] used an in vivo rat MIRI model with oxycodone 0.5 mg/kg IV (given at reperfusion) and observed reduced cardiomyocyte apoptosis (TUNEL assay) and lower CK‐MB and cTnI levels comparable to the effect of a ROCK1 pathway inhibitor. Zhao et al. [84] combined an in vivo rat model (0.3 mg/kg IV) with an in vitro H9c2 cell model, demonstrating that oxycodone significantly decreased infarct size and cardiomyocyte apoptosis while improving cardiac function (elevated ejection fraction and fractional shortening); mechanistically, oxycodone increased Bcl‐2 and p‐Akt/p‐PI3K and decreased Bax and cleaved caspase‐3, an effect abrogated by PI3K/Akt inhibition. In contrast, Ji et al. [79] focused on cardiac microvascular endothelial cells: In a rat IR model (0.5 mg/kg IV) and hypoxia/reoxygenation‐treated CMECs (using ~1–1.5 ng/mL oxycodone in vitro), oxycodone reduced infarct size and preserved endothelial integrity (upregulating ZO‐1, Occludin, and Claudin‐1) while attenuating inflammatory cytokines. Notably, Ji et al. identified the sigma‐1 receptor (SIGMAR1) as crucial: Oxycodone′s antiapoptotic and barrier‐protective effects were lost when SIGMAR1 was antagonized or silenced [79]. Jiang et al. [83] employed both an in vivo rat model (0.5 mg/kg IV) and an OGD/R injury in H9c2 cells, finding that oxycodone (including at 0.1 mM in vitro) decreased myocardial infarct size and cardiac injury markers (CK‐MB, LDH, and cTnI) and suppressed ROS generation and lipid peroxidation. Jiang′s study linked these benefits to enhanced mitochondrial stability: Oxycodone activated AMPK, upregulated SIRT1/PGC‐1*α*, preserved MMP, and its protection was blunted by an AMPK inhibitor [83]. This dosing translates to a single bolus of approximately 20–35 mg for a 70‐kg human, which appears higher than standard clinical starting doses for acute pain management (typically 5–15 mg) [121, 122]. Subsequently, the cardioprotective influences observed in animal studies use doses above typical human analgesic levels. In other fields, like neurology, projects performed underscore the important trade‐offs between ex vivo and in vivo studies [123, 124].

In vivo investigations permit the longitudinal evaluation of a drug′s impact within an integrated biological system, which is vital for comprehending its real‐time effect on a dynamic pathology such as MIRI. However, they depend on whole‐body pharmacokinetics and systemic compensatory processes [125].

Ex vivo models, such as isolated cardiomyocytes or hearts, make a highly controlled assessment of oxycodone′s direct molecular and cellular roles in the heart. This strategy allows for utilizing high, localized drug concentrations to isolate specific mechanisms—for example, the use‐dependent suppression of cardiac sodium channels (Nav1.5) observed at high micromolar levels [109].

The translation of cardioprotective influences of oxycodone is sophisticated by a key outcome; the drug′s effective concentration differs significantly between intact animal models and simplified cellular systems. Although trends of efficacy (e.g., reduced infarct size, improved cardiac function, antiapoptotic and antioxidant effects) are strong in vivo, this difference highlights an unmet need for research that directly compares outcomes and dosing among these models. Establishing an optimal dosing approach through such comparative studies is thus critical to safely realizing the clinical cardioprotective capacity of this opioid.

## 5. Conclusion

MIRI remains a significant clinical challenge with a complex pathophysiology involving inflammation, oxidative stress, and mitochondrial dysfunction. Emerging preclinical evidence positions oxycodone, a potent semisynthetic opioid, as a promising candidate for mitigating MIRI. Its cardioprotective influences are mediated through multifaceted actions, encompassing the attenuation of apoptosis via the RhoA/ROCK1 and PI3K/Akt pathways, the diminution of proinflammatory cytokines, and the alleviation of oxidative stress [79]. These mechanisms translate to promoted physiological outcomes, such as reduced infarct size, enhanced cardiac function, and diminished release of necrosis biomarkers like CK‐MB and cTnI.

A specific compelling aspect of oxycodone′s mechanism is its interaction with SIGMAR1, which seems to augment endothelial and cardiomyocyte protection. This distinct pathway, not as prominently featured as other opioids like morphine, suggests that SIGMAR1 itself could act as a novel therapeutic target. By targeting SIGMAR1 directly, it may be possible to harness the beneficial antiapoptotic and cytoprotective impacts of oxycodone′s action while circumventing its involvement with the mu‐opioid receptor, which accounts for its addictive capacity and unfavorable effects such as respiratory depression.

However, it is important to explicitly acknowledge that oxycodone is a substance with a high potential for addiction and abuse. These inherent risks, alongside other side effects and drug interactions, considerably constrain its viability as a first‐line therapeutic for MIRI in clinical practice. Hence, although oxycodone serves as a worthwhile *research tool* for addressing key cardioprotective pathways, the future of this research should not concentrate on its direct clinical utilization.

The insights gained from studying oxycodone, especially its activation of SIGMAR1 and the associated survival pathways, should inform the rational design of new, nonaddictive therapies. These could possess selective SIGMAR1 agonists or innovative compounds that replicate oxycodone′s cardioprotective impacts without the risks related to opioids.

In conclusion, while oxycodone has unraveled important mechanistic insights into fighting MIRI, its clinical exploitation for this indication is not advised. The promising path forward lies in leveraging these discoveries to develop safer, mechanism‐based alternatives. Future studies should prioritize the development and head‐to‐head comparison of these novel agents against traditional opioids to provide effective and safe cardioprotection for such cases.

## Ethics Statement

Ethical issues (including plagiarism, data fabrication, and double publication) have been completely observed by the authors.

## Disclosure

All authors read and approved the final version of the manuscript.

## Conflicts of Interest

The authors declare no conflicts of interest.

## Author Contributions

M.D.F. and B.N. contributed to the acquisition, analysis, and interpretation of data for the work and write‐up of the review article. F.R‐T. designed the framework of the manuscript.

## Funding

No funding was received for this manuscript.

## Data Availability

The datasets used and analyzed during the current study are available from the corresponding author upon reasonable request.
